# Resistance to HIV-1 infection among HIV-exposed seronegative partners in HIV-discordant couples is associated with higher frequency of CD8+ T cells expressing CD107a and b molecules

**DOI:** 10.1186/1471-2334-14-S2-P67

**Published:** 2014-05-23

**Authors:** A Padane, M Camara, M Seydi, W Jennes, AA Diallo, M Fall, PA Diaw, PS Sow, S Mboup, L Kestens, T Ndiaye Dieye

**Affiliations:** 1 Cheikh Anta Diop University, Laboratory of Immunology, CHU Le Dantec, Dakar, Senegal; 2 Cheikh Anta Diop University, Clinic of Infectious Diseases, CHU Fann, Senegal; 3 Institute of Tropical Medicine, Laboratory of Immunology, Department of Microbiology, Antwerp, Belgium

## Background

Some individuals remain persistently HIV-seronegative despite multiple high-risk exposures to the virus (HIV-exposed seronegatives or ESN). Different mechanisms may influence host resistance to HIV infection. HIV-specific CTL has been suggested to play a role in HIV-1 protection.

## Methods

Ten HIV-1 ESN in HIV-discordant couples were enrolled at the Fann University hospital, Dakar, Senegal. Thirty HIV-1 infected patients (10 HIV-1 non transmitted partners in discordant coupes and 20 HIV-1 infected partners in HIV-concordant couples) and 10 HIV-negative unexposed subjects were included as controls. Levels of CD107a and b, and CD107a/b+IFN-γ+ in CD8+ T cells sub-set were measured in fresh PBMC by flow cytometry, in the presence or absence of stimulation with SEB.

## Results

HIV-negative subjects (10 ESN subjects and 10 HIV-negative controls) showed significantly lower percentages of CD107a/b+ expression on CD8+ T-cells than did HIV-1 infected patients (2.9% vs. 11.6%, P = 0.016). Similar conclusions were reached when expression of CD107a/b+IFN-γ+ were analyzed. Interestingly, HIV-1 ESN subjects showed higher frequency of CD8+ T cells expressing CD107a and b compared with unexposed HIV-negative controls (11.6% vs. 1.3%, P = 0.018), concordant with production of intracellular IFN-γ.

## Conclusions

Taken together, our data suggest that resistance to HIV-1 infection among ESN partners in HIV-discordant couples may be associated with HIV-specific CTL responses.

